# Effects of Radix Adenophorae and Cyclosporine A on an OVA-Induced Murine Model of Asthma by Suppressing to T Cells Activity, Eosinophilia, and Bronchial Hyperresponsiveness

**DOI:** 10.1155/2008/781425

**Published:** 2008-03-18

**Authors:** Seong-Soo Roh, Seung-Hyung Kim, Young-Cheol Lee, Young-Bae Seo

**Affiliations:** ^1^Laboratory of Herbology, College of Oriental Medicine, Daegu Hanny University, Daegu 706060, South Korea; ^2^Institute of Traditional Medicine and Bioscience, Daejeon University, Daejeon 300716, South Korea; ^3^Laboratory of Herbology, College of Oriental Medicine, Sangji University, Wonju 220702, South Korea; ^4^Laboratory of Herbology, College of Oriental Medicine, Daejeon University, Daejeon 300716, South Korea

## Abstract

The present study is performed to investigate the inhibitory effects of Radix Adenophorae extract (RAE) on ovalbumin-induced asthma murine model. To study the anti-inflammatory and antiasthmatic effects of RAE, we examined the development of pulmonary eosinophilic inflammation and inhibitory effects of T cells in murine by RAE and cyclosporine A (CsA). We examined determination of airway hyperresponsiveness, flow cytometric analysis (FACS), enzyme-linked immunosorbent assay (ELISA), quantitative real time (PCR), hematoxylin-eosin staining, and Masson trichrome staining in lung tissue, lung weight, total cells, and eosinophil numbers in lung tissue. We demonstrated how RAE suppressed development on inflammation and decreased airway damage.

## 1. INTRODUCTION

Radix Adenophorae is a dry root of Umbelliferae adenophora triphilla var. japonica Hara. Radix Adenophorae has the actions of reinforcing phagocytosis of giant cell, rising leukocyte, regulating humoral and cellular immunity, antimutation, restraining adenocarcinoma cell, strengthening cardiac function, allaying a fever, and easing pain and cough [1]. It has researched that Radix Adenophorae has the chemicals such as cycloartenyl acetate, lupenone, *β*-sitosterol, taraxerone, octacosanoic acid, and praeruptorin A [2]. These chemicals are known to possess anti-inflammatory and immunomodulatory activity [3–7]. 

In modern clinical medicine, it can cure atrophic gastritis, pneumonia, thrush, lung cancer, pharyngolaryngitis, infective inflammation of trachea and lungs kinds of eosinophilic inflammation, and bronchial hyperresponsiveness [8]. And asthma is a chronic inflammatory disease of the mucosa and is associated with excess production of Th2 cytokines (IL-4, lL-5, IL-13), eosinophilic infiltration in lung. 

Th2 cells play a critical role in the pathogenesis of allergy and asthma [9] and eosinophils have a crucial role in the pathogenesis of allergic diseases and produce factors such as CCR3, CCR5, EOTAXIN 2, TGF-*β*1, and TARC. The inflammatory aspects of the disease are complex with mast cells, dendritic cells, and T and B lymphocytes, and eosinophils playing important roles. Increased eosinophils and T lymphocytes in the bronchial mucosa and bronchoalveolar lavage fluid (BALF) are distinctive features of the inflammatory response in patients with asthma and appear to correlate with the severity of the disease [10–12]. 

Clinically Radix Adenophorae has been used for treatment of asthma in Korea. But a research for this has not been accomplished yet. So to clarify the anti-inflammatory and antiasthmatic effects of Radix Adenophorae (RAE) and cyclosporine A(CsA), we examined the influence of RAE and CsA on the development of pulmonary eosinophilic inflammation in murine model of asthma. 

Therefore, we decided to investigate the effects of Radix Adenophorae on total pulmonary airflow in mice, eosinophil influx, total leukocytes number, cell surface markers by FACS, and cytokines production in BALF by ELISA were determined. 

## 2. MATERIALS AND METHODS

Plant material and Radix Adenophorae of extracts. The sample of the Radix Adenophorae was purchased from Oriental Medical Hospital (Daejeon, South Korea) in 2007. The plant was identified by Professor Young-Bae Seo and avoucher specimens (Adenophorae) are deposited in our laboratory. Plant material (200 g) was extracted three times with distilled H_2_O (or distilled water). Then, the extract was filtered and evaporated on a rotary evaporator (BUCHI, Switzerland), dried by a freeze drier (EYELA FDU-540, Tokyo, Japan) to yield the extract of Adenophorae Radix (20 g). The yield (*w/w*) of the extract was about 10%.

**Animals.**

Seven-eight-week-old female C57bl/6 mice were obtained at Daehan Biolink Co. Ltd. (Eumsung, South Korea). All animal procedures were conducted in accordance with the guidelines of the Institutional Animal Care and Use Committee, Korea Research Institute of Bioscience and Biotechnology (Daejeon, South Korea). We were divided into five groups and each group had six mice.

**Isolation CD4^+^ T cells.**

Splenocytes were isolated from naive C57bl/6 mice. Cells were enriched for CD4^+^ cell populations by first staining the cells with anti-CD4 (BD PharMingen, Calif, USA). CD25-cells were isolated from this population by first staining with fluorescein isothiocyanate (FITC)—conjugated anti-CD25 mAb (BD PharMingen) followed by incubation with magnetic-activated cell sorting anti-FITC beads (Miltenyi Biotec, Auburn, Calif, USA). CD4^+^ T cells were selected on a (CS) column, and the flow-through was collected as CD4^+^ T cells. Isolated cells were activated by overnight incubation on 24-well plates coated with 1 *μ*g/mL anti-CD3 (datasheet: MCA500GA; description: Rat antimouse CD3; specificity: CD3; format: purified; product type: monoclonal antibody; clone: KT3; isotype: IgG2a; quantity: 0.25 mg, AbD Serotec.), 1 *μ*g/mL anti-CD28 (datasheet: MCA1363; description: Hamster antimouse CD28; specificity: CD28; format: purified; product type: monoclonal antibody; clone: 37.51.1; isotype: IgG; quantity: 0.2 mg) and with RAE (100, 50, 10  *μ*g/mL) added to RPMI medium supplemented with 1 unit/mL penicillin, 1 *μ*g/mL streptomycin, 20 mM L-glutamine, 50 mM-mercaptoethanol, and 5% FBS for 48 hours after stimulation.

**Cytokine measurements.**

After 48-hours cultures, samples were centrifuged at 2 000 rpm for 10 minutes, and the supernatants were stored at −80°C. Mouse IL-4, IL-5, IL-13, and IFN-*γ* were quantified using ELISA kits (BioSource International, Camarillo, Calif, USA) according to the manufacturer’s protocols.

**Digestion of pulmonary tissue and cells preparations.**

Single-cell suspensions from lung tissues and BALF were isolated by mechanical disruption in RPMI 1640 medium supplemented with 2 mM L-glutamine, 100 U/mL penicillin, 100 mL streptomycin, 50 mM 2-mercaptoethanol, 20 mM HEPES, and 2% heat-inactivated fetal bovine serum (FBS, GIBCO, Grand Island, NY, USA). Briefly, lungs were subsequently removed from thoracic cavity. After mincing using sterile scalpels, tissue was incubated in PBS containing 1 *μ*g/mL collagenase IV and 2 *μ*g/mL dispase II for 40 minutes at 37°C in a sterile polypropylene tube. After incubation, lung tissue was vigorously pipetted up and down to further dissolve remaining tissue clumps and then filtered using 70 *μ*m cellstrainer (Falcon, Le Pont de Claix, France). Total cells were counted manually in a hemocytometer chamber (Fisher). A number of 2 – 4 × 10^3^ cells were spun onto glass slides (Cytospin centrifuge, Cellspin, Hanil, South Korea) (400 xg for 4 minutes). Differential count was made according to standard morphologic criteria.

**OVA sensitization and inhalation.**

By modified protocol as previously described [13, 14], OVA (500  *μ*g/mL) in PBS was mixed with equal volumes of 10% (w/v) aluminum potassium sulfate (alum; Sigma-Aldrich) in distilled water. Then incubated for 60 minutes at RT after adjustment to pH 6.5 using 10 N NaOH, and centrifuged at 750 Xg for 5 minutes. OVA/alum pellet was resuspened to the original volume in distilled water. The experimental animals were sensitized by intraperitoneal (i.p) injection of OVA/alum {500 *μg*/ml, 0.2*μg* alum-precipitated Ag containing 100 *μ*g of OVA (Sigma-Aldrich, Seoul, South Korea) bound to 4 mg of aluminum hydroxide (Sigma-Aldrich) in PBS} with the amount of 200 *μ*g on day 0, and 100 mL on days 7 and 14. And the mice were challenged with an intratracheal (i.t) injection of 100 mL OVA/alum (500 *μ*g/mL) on day 21, and underwent an aerosol challenge with OVA/alum for 30 min/day on 5 days/week for 8 weeks (at a flow rate of 250 L/min, 2.5% ovalbumin in normal saline). Seven days after the second sensitization, mice were exposed to aerosolized ovalbumin for 30 min/day on 5 days/week for 8 weeks (at a flow rate of 250 L/min, 2.5% ovalbumin in normal saline). The RAE (450, 45 mg/kg) in solution form were orally administered five times a week and CsA (10 mg/kg) injection three times a week for the last 8 weeks once 0.1 mL. One day after the last of the OVA exposures, samples (BALF, lung cells, and blood) were collected.

**BALF.**

To evaluate airway inflammation, we experimented the accumulation of eosinophils in BALF. Mice were sacrificed with an intraperitoneal injection of sodium pentoparbitone (100 mg/kg). The trachea was cannulated and the left bronchi were tied for histological experiment.

Immediately after sacrifice, cells in the lungs were recovered by flushing 1 mL of BALF (1 mM EDTA, 10% FBS, PBS) into the lungs via the trachea. Total cell counts were determined and 100 mL of fluid were cytospin onto glass slides using a cytospin centrifuge (Cellspin, Hanil, South Korea) ( 400 ×g for 4 minutes). Differential cell counts were performed after staining with Diff-Quik Stain Set (Baxter Healthcare Corporation, Miami, Fla, USA). The supernatant of BALF was stored at –25°C for determination of cytokines.

**Determination of airway hyperresponsiveness.**

Total pulmonary airflow in mice was estimated in a modification of the method [15, 16]. Buxco system (Biosystem XA; Buxco Electronics Inc., Troy, Conn, USA) was used to evaluate the extent of airway constriction in different groups of mice following the protocol described previously.

Penh is equal to Pause *x* PIF/PEF, where Pause = (Te − Tr)/Tr (PIF: peak inspiratory flow; PEF: peak expiratory flow; Te: expiratory time; Tr: relaxation time). In this experiment, the mice were aerosolized ovalbumin for 30 min/day on 3 days/week for 12 weeks. Twenty four hours after final inhalation, the mice were given aerosolized normal saline, 50 *μ*g/mL methacholine (Sigma-Aldrich). And then airway reactivity was monitored for 1, 3, 10, and 30 minutes, serially. Differences of Penh value between groups were evaluated by means of student’s *t*-test.

**Antibodies and flow cytometric analysis.**

All antibodies (CD3, CD4, CD8, CD69, CCR3, CD11b, Gr-1, IgE, and B220) for flow cytometric analysis were purchased from Becton Dickinson (BD) PharMingen (San Diego, Calif, USA). Cells from lung tissues and BALF were stained with the indicated antibodies in staining buffer (PBS containing 1% FBS and 0.01% NaN_3_) for 10 minutes on ice, and analyzed by two-color flow cytometry on a FACScan using CellQuest software (BD Biosciences, Mountain View, Calif, USA).

**Enzyme-linked immimosorbent assay.**

Interleukin (IL-4, IL-5, IL-13, IL-I0), IgE production from BALF and histamine release in serum of the indicated mice (*n* = 6) was measured by ELISA according to the manufacturer's instruction on a monoclonal antibody-based mouse ELISA kit. All data represent the standard deviation of at least three different determinants and were compared using Student's *t*-test.

**Quantitative real-time PCR.**

To study antiasthmatic effects of Radix Adenophorae extract on cytokine gene expression from lung cells, quantitative real-time PCR was performed after quantitative normalization for each gene by a densitometry using *β*-actin gene expression. Briefly, total cellular RNA was extracted from the lung by the phenol-chloroform-based method (RNAsolB: Tel-Test Co. Inc., Friendswood, Tex, USA) according to the manufacturer's instructions. cDNA synthesis from 3 *μ*g of total RNA was carried out using a ReverTraAce-a-cDNA Synthesis kit (Toyobo Co., Ltd., Osaka, Japan) according to the manufacturer’s instructions. Real-time quantitative PCR was performed using the Applied Biosystems 7500 Fast Real-Time PCR system (Applied Biosystems, Foster City, Calif, USA) with the following primers.

The primer sequences are as follows: mouse eotaxin 2, 5^*′*^-CCTGGACCAAAAACTCCAAA-3^*′*^ and 5^*′*^-GCGACTGGTGCTGATATTC-3^*′*^ CCR3, 5^*′*^-CCCGAACTGTGACITTTGCT-3^*′*^ and 5^*′*^-CCTCTGTATAGCGAGGACTG-3^*′*^ IL-13, 5^*′*^-ATGCCCAACAAAGCAGAGAC-3^*′*^ and 5^*′*^-TGAGAGAACCAGGGAGCTGT-3^*′*^ TNF- *α*, 5^*′*^-TGGGAGGAAGGGGTCTAAG-3^*′*^ and 5^*′*^-ACCTACGACGTGGGCTACAG-3^*′*^ IL-10, 5^*′*^-AAGCAGCCTTGCAGAAAAGA-3^*′*^ and 5^*′*^-TGGGAAGTGGGTGCAGTTAT-3^*′*^ TGF- *β*1, 5^*′*^-TGGAGCAACATGTGGAACTC-3^*′*^ and 5^*′*^-CTGCCGTACAACTCCAGTGA-3^*′*^ TARC, 5^*′*^-CAGGGATGCCATCGTGTTTCT-3^*′*^ and 5^*′*^-GGTCACAGGCCGCTTTATGTT-3^*′*^ *β*-actin, 5^*′*^-TGGAATCCTGGTCCATGAAAC-3^*′*^ and 5^*′*^-GTCACAGTCAGCTGTATAGGG-3^*′*^.

Proinflammatory gene expression was analyzed with SYBR Green PCR Mastermix (ABl) and a final concentration of 200 nM primers, using *β*-actin as the internal standard. The following PCR profile was used: 2 minutes at 50°C, 10 minutes at 94°C, and 40 cycles of 1 minute at 94°C, and 1 minute at 60°C. The amount of SYBR Green was measured at the end of each cycle. The cycle number at which the emission intensity of the sample rises above the baseline is referred as to the relative quantitative (RQ) and is proportional to the target concentration. Real-time PCR was performed in duplicate and analyzed by applied Biosystems 7500 Fast Real-Time PCR system manual (threshold: 0.05; baseline: 6–15 cycles). To generate the standard curves for Proinflammatory cytokine and *β*-actin, serially diluted cDNA (1/1-1/16) was prepared and real-time PCR was performed as above. Relative quantitative (RQ) evaluations by RQ-PCR were expressed at various samples.

**H*&*E and M-T staining in murine OVA-induced asthma lung tissue.**

Three experimental groups were treated with different concentrations of RAE five times a week and CsA three times a week for the later 8 weeks. At the end of the experiment, the mice lung sections were stained with H*&*E and M-T and analyzed histologically by modified protocol as previously described [17, 18].

**Statiscal analysis.**

For statistical analysis of data, *P* values were analyzed using a student’s *t*-test software program (Startview 5.1; Abacus Concepts, Berkeley, Calif, USA). Results were considered statistically significant when *P* values were ^*^ *p* < .05, ^**^ *p* < .01, and ^***^ *p* < .001.

## 3. RESULTS AND DISCUSSION

First, we examined how RAE made an effect toward CD4^+^ T cells in vitro. Splenocytes were isolated from naive C57bl/6 mice. CD4^+^ T cells were selected on a CS column, and the flow-through was collected as CD4^+^ T cells. Isolated cells were activated by overnight incubation on 24-well plates coated with 1 *μ*g/mL anti-CD3, 1 *μ*g/mL anti-CD28, and with RAE (100, 50, 10 *μ*g/mL) and CsA (Cyclosporine A 10 *μ*g/mL) added to RPMI medium supplemented with 1 unit/mL penicillin, 1 *μ*g/mL streptomycin, 20 mM L-glutamine, 50 mM-mercaptoethanol, and 5% FBS for 48 hours after stimulation. After 48-hour culture, samples were centrifuged at 2 000 rpm for 10 minutes. Mouse IL-4, IL-5, IL-13, and IFN-*γ*. The levels of cytokines were quantified using ELISA kits according to the manufacturer’s protocols. As you see Table 1, RAE decreased IL-4, IL-5, and IL-13 cytokines, It has been suggested that RAE may be has suppressing activities of CD4^+^ T cells, eosinophils inducing asthmatic inflammations.

The results expressed the mean ± S.E (*N* = 6). Statistically significant value compared with control group data by *t*-test (^***^ *p* < .001, ^**^ *p* < .01, ^*^ *p* < .05.

**Figure 1:**
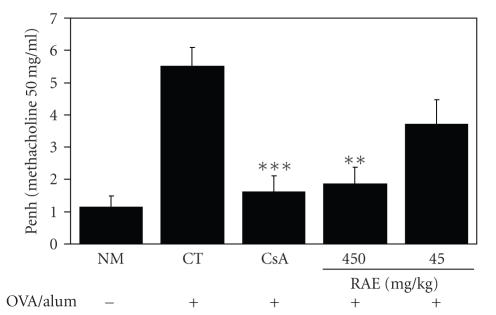
Effects of RAE and CsA on methacholine-induced AHR in the sensitization (NM: normal C57BL/6 mice; CT: OVA-induced asthma mice (control); CsA: OVA-induced asthma mice treated with cyclosporine A (10 mg/kg); RAE 450 mg/kg: OVA-induced asthma mice treated with RAE (450 mg/kg); RAE 45 mg/kg: OVA-induced asthma mice treated with RAE (45 mg/kg)).

To evaluate the effects of RAE and CsA on airway hyperresponsiveness, total pulmonary airflow in mice was estimated in murine model of asthma. Penh was measured by Buxco system on day 1 after final inhalation and then immediately samples were collected. Exposure of animals to aerosolized OVA resulted in increased airway hyperresponsiveness (AHR) compared with that of animals receiving PBS only (Figure 1). As shown in Figure 1, relative to animals sensitized with OVA (control group), RAE-(450 mg/kg) and CsA-(10 mg/kg) treated groups showed a significant ( ^**^ *p* < .01, ^***^ *p* < .001) decrease in methacholine-induced AHR. But RAE (45 mg/kg)group did not show significant decrease in Penh value. This was accompanied by changes in the lung and BAL total cells counts (Figure 4). Therefore, above results indicate that RAE (450 mg/kg) and CsA have inhibitory effects on AHR. 

Statistically significant, value compared with control by *t*-test (^*^ *p* < .05, ^**^ *p* < .01). 

**Figure 2:**
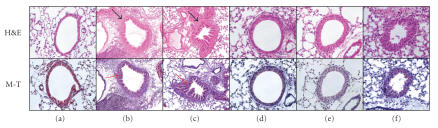
Effect of RAE on histology of lung tissue (H*&*E and Masson trichrome staining) in lung tissues of OVA-induced asthma murine ((a): normal C57BL/6 mice; (b): OVA-induced asthma mice (Control 1); (c): OVA-induced asthma mice (Control 2); (d): OVA-induced asthma mice treated with cyclosporine A (10 mg/kg); (e): OVA-induced asthma mice treated with RAE (450 mg/kg); (f): OVA-induced asthma mice treated with RAE (45 mg/kg)).

**Figure 3:**
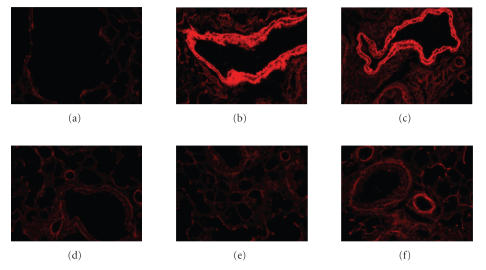
Effect of RAE on immunohistochemistry of lung tissue (Congo red stain) in lung tissues of OVA-induced asthma murine ((a): normal C57bl/6 mice; (b) and (c): OVA-induced asthma mice (control); (d): OVA-induced asthma mice treated with cyclosporine A (10 mg/kg); (e): OVA-induced asthma mice treated with RAE (450 mg/kg); (f): OVA-induced asthma mice treated with RAE (45 mg/kg)).

To clarify the efficacy of RAE on lung cells of murine asthma model, the left lungs were histologically examined 24 hours after the final antigen challenge. Histological analyses of lungs from PBS-exposed sensitized mice showed normal lung histology (Figures 2(a), 3(a)). In contrast, similar to the BALF study, histological sections of lung tissue from OVA-exposed mice exhibited airway inflammation and infiltrating eosinophils were chiefly observed in the peribronchial regions of the lung (Figures 2(b), 2(c), 3(b), 3(c)). While on the other hand, exhibition of airway inflammation was decreased in histological sections of lung tissue from RAE and CsA (Figures 2(d), 3(d)), RAE 450 mg/kg (Figures 2(e), 3(e)), and RAE 45 mg/kg-treated mice (Figures 2(f), 3(f)). The lung tissue of CsA and RAE treatments on mice group showed much less eosinophils, leukocytes, and collagen accumulating compared with that of OVA-induced mice group. 

**Figure 4:**
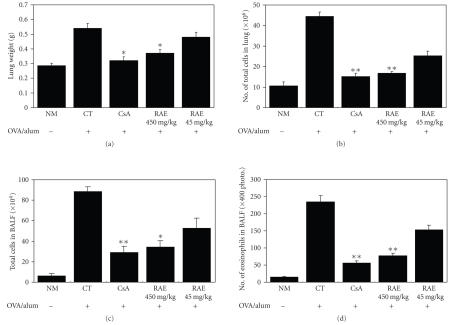
Effects of RAE on lung weights and total lung cells in OVA-induced asthma murine (NM: normal C57BL/6 mice; CT: OVA-induced asthma mice (control); CsA: OVA-induced asthma mice treated with cyclosporine A (10 mg/kg); RAE 450 mg/kg: OVA-induced asthma mice treated with RAE (450 mg/kg); RAE 45 mg/kg: OVA-induced asthma mice treated with RAE (45 mg/kg)).

The number of BAL eosinophils was sequentially increased after two or three consecutive days of OVA exposure. To evaluate the effect of RAE and CsA on the recruitment of cells to the airway, we investigated the lung weight, total lung cell, total leukocytes, and eosinophils in BALF. In RAE and CsA-treated groups, total lung weight was significantly reduced compared with OVA-exposed control group, and the number of total leukocytes in BALF, total lung cells, and eosinophils were also significantly reduced compared to those in the control group (Figure 4). 

C57BL/6 mice were injected, inhaled, and sprayed with ovalbumin (OVA) for 8 weeks (five times a week) for asthma induction. Two experimental groups were treated with different concentrations of Radix Adenophorae extract (RAE) for the later 8 weeks (five times a week). At the end of the experiment, the mice lungs were removed and weighted. 

The cell surface expression of CCR3 was analyzed by flow cytometry. To detect CCR3 expression on lung, lymph node, BALF, and spleen cells, two-color immune-staining was performed using FITC-conjugated anti-CCR3 with PE-conjugated anti-CD3 mAb as described in materials and methods. 

**Figure 5:**
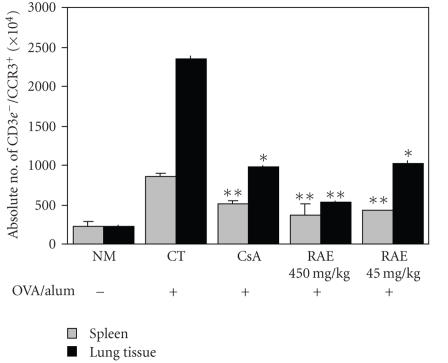
Effect of RAE on CD3e^−^/CCR3^+^ population in lung tissues of OVA-induced asthma murine (NM: normal C57BL/6 mice; CT: OVA-induced asthma mice (control); CsA: OVA-induced asthma mice treated with cyclosporine A (10 mg/kg); RAE 450 mg/kg: OVA-induced asthma mice treated with RAE (450 mg/kg); RAE 45 mg/kg: OVA-induced asthma mice treated with RAE (45 mg/kg)).

**Figure 6:**
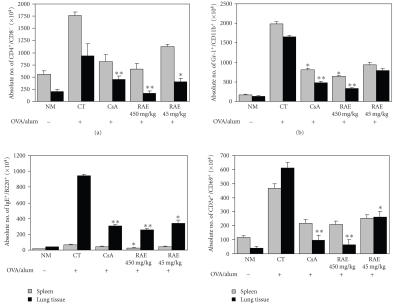
Effects of RAE on CD4^+^/CD8^−^, GR-1^+^/CD11b^+^, IgE^+^/B220^+^, and CD3e^+^/CD69^+^ cells absolute number in lung and spleen tissues of OVA-induced asthma murine (NM: normal C57BL/6 mice; CT: OVA-induced asthma mice (control); CsA: OVA-induced asthma mice treated with cyclosporine A (10 mg/kg); RAE 450 mg/kg: OVA-induced asthma mice treated with RAE (450 mg/kg); RAE 45 mg/kg: OVA-induced asthma mice treated with RAE (45 mg/kg)).

To evaluate the efficacy of RAE and CsA treatments on granulocytes population and CCR3 expression, we compared the effects of RAE and CsA on intracellular CCR3 expression in OVA-induced asthma lung cells by using flow cytometry. The percentages of CCR3-positive cells were decreased when compared with control group (^**^ *p* < .01, Figure 5). Results obtained with FACS were also confirmed by real-time PCR, as the relative quantitiveness RQ of mRNA expression in lung cells expressing CCR3 was significantly decreased in cells treated with RAE and CsA when compared with control group (Table 3). Moreover, RAE and CsA treated group with OVA resulted in significant reductions Gr-1^+^/CD11b^+^ cells in lung ^**^ *p* < .01) (Figure 6). 

C57BL/6 mice were injected, inhaled, and sprayed with OVA for 8 weeks (five times a week) for asthma induction. Two experimental groups were treated with different concentrations of RAE for the later 8 weeks (five times a week). At the end of the experiment, the mice lungs were removed and analyzed by flow cytometer.

Effects of RAE on leukocyte subsets in lungs and BALF were marked with change in numbers of CD4^+^/CD8^−^ T cells (Th2 cells). CD3 ^+^/CD69^+^ (early activated T cells) IgE^+^/B220^+^ B cells in a murine model of asthma compared to control group, and the deficits in CD4^+^/CD8^−^ T cells were accompanied by concurrent decreases in absolute number of CD3^+^/CD69^+^, IgE^+^/B220^+^ B cells (Table 2, Figure 6). RAE and CsA groups treated with OVA resulted in further significant reductions in CD4^+^/CD8^−^ T cells (^**^ *p* < .01) and IgE^+^/B220^+^ B cells (^**^ *p* < .01) in BALF and lung tissues significantly were decreased and CD4^+^/CD8^−^ T cells and CD3^+^/CD69^+^ cells (^**^ *p* < .01) in lung tissues significantly were also decreased (Table 2, Figure 6). 

Each point represents the mean ± S.E of 6 mice. Statistically significant, value compared with control by *t*-test ^*^ *p* < .05, ^**^ *p* < .01) (LN^1^: Lymph node; B A^2^: Bronchoalveolar Lavage).

**Figure 7:**
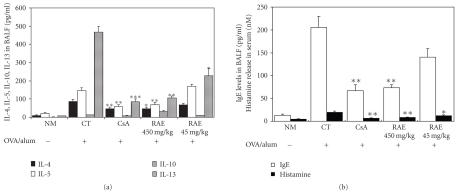
Effect of RAE on cytokines, immunoglobulin E levels in BALF and histamine release in serum (NM: normal C57BL/6 mice; CT: OVA-induced asthma mice (control); CsA: OVA-induced asthma mice treated with cyclosporine A (10 mg/kg); RAE 450 mg/kg: OVA-induced asthma mice treated with RAE (450 mg/kg); RAE 450 mg/kg: OVA-induced asthma mice treated with RAE (450 mg/kg)).

To study whether RAE was related to inflammatory cytokine production, BALF supernatants were collected, and the productions of IL-4, IL-5, IL-10, IL-13, IgE, histamine were analyzed by ELISA. Asshown in Figure 7, IL-4, IL-5, IL-13, IgE productions in BALF and histamine production in serum were suppressed by RAE and CsA. Both IL-4 and IL-13 levels were significantly reduced in RAE- and CsA-treated groups of mice. 

Because IgE levels in BALF are dependent upon IL-4, IL-5, IL-13 and may be considered an additional index of Th2 cytokine secretion, we measured IgE in BALF from mice in all groups. We found that IgE levels in BALF from OVA-induced murine model of asthma were significantly increased compared with normal groups. RAE and CsA treatments of these mice significantly inhibited the production of IgE. These results support the conclusion that RAE and CsA suppressed the generation of a Th2-type immune response and activity of mast cells in this animal model of asthma. 

To investigate the effect of RAE on mRNA expression in lung tissue, total cellular RNA was extracted from lung cells treated with or without RAE in the presence or absence of OVA sensitization and inhalation. As shown in Table 3, the mRNA for eotaxin2, CCR3, IL-13, IL-10, TARC, and TNF-*α* was detectable in the lung cells treated with PBS only (NM), OVA (CT), CsA (10 mg/kg), and RAE (450, 45 mg/kg), respectively (Table 3). 

C57BL/6 mice were injected, inhaled and, sprayed with OVA for 8 weeks (five times a week) for asthma induction. Two experimental groups were treated with different concentrations of Radix Adenophorae extract (RAE) for the later 8 weeks (five times a week). At the end of the experiment, the mice lungs were removed and analyzed by real-time PCR. 

PCR products for eotaxin2, CCR3, IL-13, TARC, and TNF- *α* amplified from lung cell RNA preparations were decreased in the RAE-treated groups compared with control groups. Furthermore, the RAE had little effects on IL-10 mRNA expression in lung cells (Table 3). The results demonstrated that RAE and CsA significantly affected eotaxin2, CCR3, IL-13, TARC, and TNF- *α* mRNAs expression but not other cytokines in lung cells. This was accompanied by changes in the eosinophil influx (Figure 5), BAL cytokines (IL-13) production (Figure 7) in some degree. 

C57BL/6 mice were injected, inhaled, and sprayed with OVA for 8 weeks (five times a week) for asthma induction. Two experimental groups were treated with different concentrations of Radix Adenophorae extract (RAE) for the later 8 weeks (five times a week). At the end of the experiment, the BALF were collected and cytokine productions were analyzed by ELISA. 

Bronchial asthma is a chronic inflammatory disorder of the airways characterized by variable airway obstruction, airway eosinophilic inflammation, and AHR [19]. Persistent inflammation of bronchial mucosa, mainly characterized by eosinophilic infiltration, is considered as an essential process in the pathogenesis of asthma [20, 21]. 

Asthma is a chronic lung disease characterized by allergen-induced airway inflammation and orchestrated by Th2 cells [22]. RAE is one of the well known herbs used in oriental medicine for treatment of anti-inflammatory and many allergic diseases. 

Anti-inflammatory effects of RAE on the development of OVA-induced eosinophilia and hyperresponsiveness in murine model of asthma have not been fully investigated in vivo. 

Immunomodulators such as cyclosporine A (CsA) inhibit single allergen-induced allergic inflammation such as eosinophilic, lymphocytic infiltration, and mRNA expression for IL-4 and IL-5 [23]. In this study, the RAE could modulate the immune responses of BALF cells and lung cells in OVA-induced murine model of asthma. Hence, the suppressive activity of RAE and CsA on inflammatory cytokine productions might have important implications with regard to RAE therapeutic activity in asthma and pulmonary inflammation. The main question to be addressed is whether RAE is involved in the pathology of airway inflammation and asthma. In the present study, we examined lung cells inflammation and BALF analyses. 

Airway allergen challenge causes cellular inflammation in the airways, which is dominated by eosinophils in both humans and mice [24]. It has been suggested that eosinophils contribute to several of the clinical features of allergic asthma, including tissue damage and AHR [25]. 

We measured the effects of RAE on methacholine-induced AHR in the sensitization. As shown in Figure 1, relative to animals sensitized with OVA (control group), RAE-(450 mg/kg) and CsA-(10 mg/kg) treated groups showed a significant decrease in methacholine-induced AHR. This was accompanied by changes in the lung and BAL total cells counts (Figure 4). Therefore, above results indicate that RAE (450 mg/kg) and CsA have inhibitory effects on airway hyperresponsiveness. 

In aspect of histological analyses of sections from OVA-induced asthma model mice, the lung tissue of CsA and RAE treatments of mice group showed much less eosinophils, leukocytes, and collagen accumulating compared with that of OVA-induced mice group (Figures 2, 3). 

To evaluate the effect of RAE and CsA on the recruitment of cells to the airway, we investigated the lung weight, total lung cell, total leukocytes, and eosinophils in BALF. In groups treated with RAE and CsA, total lung weight was significantly reduced compared with OVA-exposed control group, and the number of total leukocytes in BALF, total lung cells, and eosinophils was also significantly reduced compared to those in the control group (Figure 4). These results indicate that RAE (450 mg/kg) and CsA have inhibitory effects on eosinophils and leukocytes infiltration into the lungs. 

Antigen-activatedCD4^+^ T cells have been shown to induce many of the characteristic features of asthma, including the secretion of cytokines such as IL-4, IL-5, and IL-13, which regulate mucus production, inflammation, and adhesion molecules [26–28]. 

High levels of interleukin-4 and interleukin-5 are related to clinical measures of disease severity [29, 30]; IL-5 has been shown to mediate the recruitment of eosinophils [31]. The reduction in BAL IL-13 levels would suggest a beneficial therapeutic effect via decreased mucus production [32]. 

To evaluate the efficacy of RAE and CsA treatments on granulocytes population and CCR3 expression, we compared the effects of RAE and CsA on intracellular CCR3 expression in OVA-induced asthma lung cells by using flow cytometry. The percentages of CCR3-positive cells were decreased when compared with control group (Figure 5). Results obtained with FACS were also confirmed by real-time PCR, as the relative quantitiveness RQ of mRNA gene expression in lung cells expressing CCR3 was significantly decreased in cells treated with RAE and CsA when compared with control group (Table 3). Our results indicate that RAE could decrease CCR3 expression of BALF cells (especially eosinophils), data of which are compatible with airway eosinophil influx and hyperresponsiveness. 

CD11b expression on the surface of circulating eosino-phils is significantly elevated in various allergic disorders, including atopic dermatitis and bronchial asthma [33]. We observed that inhalation challenge with RAE and CsA administration resulted in a decrease in airway CD11b^+^ macrophage, Gr-1 granulocytes when compared with that observed after OVA challenge only. RAE and CsA groups treated with OVA resulted in significant reductions Gr-1^+^/CD11b cells in lung and lymph nodes (Table 2, Figure 6). Our results show that RAE and CsA downregulate CCR3 expression and CD4^+^CD8^−^ T cells, CD3^+^ CD69^+^, IgE^+^ B220^+^ B cells in BALF and lung cells (Table 2, Figure 6). 

In study, various cytokines production of BALF cells from OVA-induced control subjects have been enhanced, but RAE- and CsA-treated subjects significantly reduced. We suggest that the inhibitory mechanism of RAE and CsA on IL-4, IL-5, IL-10, and IL-13 production may have involved reduced production of IL-4, IL-5, IL-13, IgE and this would suppress the eosinophils influx in airway and then more factors synthesized by RAE and CsA treated in BALF such as IgE were decreased (Figure 7). This was accompanied by changes in the BAL eosinophils counts (Figure 4) and mRNA expression in lung tissue (Table 3). 

 RAE decreased IL-4, IL-5, and IL-13 levels in BALF and histamine release in serum significantly (Figure 7), and mRNA expression of IL-13 in the lung as well (Table 3). The lung histology in our study (Figures 2, 3) showed that RAE reduced the eosinophils in lung tissue, and this result may be due to the decreased IL-4 level which caused reduction in rolling and adhesion of eosinophils. Based on these results, it is inferred that RAE may have an antiallergic effect on allergic bronchial asthma via suppressing IL-4 secretion, and in consequence, inhibiting IgE secretion from B cells, and reduced IL-4 may reduce eosinophils infiltration into the lungs via reducing the rolling on and adhesion of circulating eosinophils to the endothelial cells. 

IL-5 is a Th2-type cytokine that promotes the recruitment and activation of eosinophils. IL-5 stimulates the release of chemical mediators from the eosinophils [34]. Therefore, IL-5 has been implicated in the pathogenesis of allergic diseases [35], and many studies have focused IL-5 to be a viable target for the treatment of asthma and allergic diseases [36]. 

In our study, RAE significantly reduced IL-5 levels in BALF (Figure 7) as well as mRNA expression of IL-13 in lung (Table 3). Like this, RAE reduced eosinophils infiltration and activation in OVA-induced asthmatic mice. We considered that this action may be due to inhibiting IL-13, IL-5 secretion from Th2 cells. 

These results indicate that RAE may suppress the excess activity of T cells and Th2 cytokines, which are implicated in the pathogenesis of allergic asthma, and consequently regulate the Th1/Th2 imbalance of the immune system in allergic asthma. From these results, we hypothesize that RAE has an immunoregulatory function between Th1 and Th2, and suppressive effect on excessive Th2 cytokines. 

Th2 cytokines as well as elevated IgE levels are associated with the development of airway hyperreactivity. Especially, IL-13 has been shown to mediate the airway hyperresponsiveness in an allergic lung in several studies [37]. 

Furthermore, mRNA expressions of these cytokines in lung were reduced remarkably by RAE (Table 2). From these results, we presume that RAE may suppress the airway hyperresponsiveness by way of reducing these cytokines. 

Cytokines and chemokines produced by Bronchial epithelial cells include IL-6, IL-8, G-CSF, GM-CSF, RANTES, eotaxin, and TARC. Proinflammatory cytokines IL-1 and TNF-alpha generally up-regulate expression and release these cytokines/chemokines [38]. 

The bronchial epithelium of asthmatics and normal subjects expressed TARC protein, and the asthmatics showed more intense expression than the normal subjects. And combination of TNF-alpha and IL-4 induced expression of TARC protein. Furthermore, expressions of TARC protein and mRNA were almost completely inhibited by glucocorticoids. The airway epithelium represents an important source of TARC, which potentially plays a role via a paracrine mechanism in the development of allergic respiratory diseases [39].

Our study shows that eotaxin2, TNF-*α*, and TARC mDNA gene expression in lung cells were significantly decreased in cells treated with RAE and CsA when compared with control group (Table 3). Our results indicate that RAE may have effects of antibronchial injury, data of which are compatible with airway eosinophils influx and hyperresponsiveness. So histological analyses of sections from OVA-induced asthma model mice, the lung tissue of CsA and RAE treatments of mice group showed much less eosinophils, leukocytes, and collagen accumulating compared with that of OVA-induced mice group (Figures 2, 3). 

In summary, RAE and CsA have profound effects on airway inflammation and AHR in a murine model of asthma through suppression of IL-13, IL-5, IgE, eosinophils CCR3 expression, and CD11b cells expression. The therapeutic activity of RAE on asthma in oriental medicine may be partly related tothe following. (a) Immunomodulatory agents contained in RAE reduce Th2 cells immune responses. (b) Reduced production of IgE will make a marked decrease for asthma attack occurring in individuals. (c) RAE may have an effect of suppressing eosinophils activity and decrease airway damage.

## Figures and Tables

**Table 1:** Effects of RAE on cytokines in CD4^+^ t cells isolated from Naive C57bl/6 Mice (NM: Normal C57BL/6 mice; CT: a-CD3/CD28^+^ CD4^+^ T cells; CsA: a-CD3/CD28^+^ CD4^+^ T cells + CsA; RAE 100: a-CD3/CD28^+^ CD4^+^ T cells + RAE (100 *μ*g/mL); RAE 50: a-CD3/CD28^+^ CD4^+^ T cells + RAE (50 *μ*g/mL); RAE 10: a-CD3/CD28^+^ CD4^+^ T cells + RAE (10 *μ*g/mL)).

	IFN-gamma (pg/mL)	IL-4 (pg/mL)	IL-5 (pg/mL)	IL-13 (pg/mL)
NM	1023.8 ± 39.3	693.8 ± 109.9	1.0 ± 0.2	15.2 ± 1.0
CT	1452.5 ± 62.4	4681 ± 303.5	54.5 ± 5.1	515.5 ± 18.7
CsA	809 ± 32.2^***^	537 ± 84.3^***^	3.9 ± 0.4^***^	15.4 ± 0.7^***^
RAE 100	2028.9 ± 192.9^*^	2859.5 ± 198.7^**^	21.7 ± 1.4^***^	316.2 ± 32.6^***^
RAE 50	1529.1 ± 159.9	3114.1 ± 76.2^**^	28.3 ± 2.7^**^	394.4 ± 21.7^**^
RAE 10	1335.4 ± 55.6	3597.2 ± 434.7	56.7 ± 4.6	496.5 ± 7.2

**Table 2:** Effects of RAE on CD4^+^/CD8^−^, GR-1^+^/CD11b^+^, IgE^+^/B220^+^, and CD3e^+^/CD69^+^ cells absolute number in lymph nodes and BAL fluid of OVA-induced asthma murine.

Cell phenotype	Organ	Normal C57BL/6	OVA-induced asthma mice (Absolute no.)			
			Control	CsA	RAE (450)	RAE (45)
CD4^+^/CD8^−^ (×10^4^ cells)	LN^1^	221.9 ± 34.9	232.7 ± 12.8	150.3 ± 41.1^**^	193.95 ± 10.1^*^	244.7 ± 32.2
	BA^2^	3.9 ± 0.6	33.6 ± 0.8	13.2 ± 3.1^**^	11.3 ± 0.1^**^	27.7 ± 1.5^*^
Gr-^1^/CD11b^+^ (×10^4^ cells)	LN^1^	25.6 ± 0.5	73.6 ± 13.8	45.0 ± 11.9^**^	33.7 ± 1.0^**^	45.6 ± 8.6^*^
IgE^+^/B220^+^ (×10^4^ cells)	LN^1^	3.6 ± 0.1	6.6 ± 1.2	3.6 ± 1.1	2.1 ± 0.3^*^	3.0 ± 0.9
	BA^2^	0.3 ± 0.01	2.6 ± 0.07	0.5 ± 0.03^**^	0.5 ± 0.02^**^	1.1 ± 0.05^*^
CD3^+^/CD69^+^ (×10^4^ cells)	LN^1^	32.5 ± 3.0	54.2 ± 1.4	35.6 ± 8.1	36.6 ± 1.9	47.6 ± 7.1

**Table 3:** Effect of RAE on Eotaxin2, CCR3, IL-13, IL-10, TNF-*α*, and TARC mRNA expression in lung tissue of OVA-induced murine model of asthma (NM: normal C57BL/6 mice; CT: OVA-induced asthma mice (control); CsA: OVA-induced asthma mice treated with cyclosporine A (10 mg/kg); RAE 45 mg/kg: OVA-induced asthma mice treated with RAE (450 mg/kg); RAE 450 mg/kg: OVA-induced asthma mice treated with RAE (450 mg/kg)).

OVA/alum	Group	Exotaxin2	CCR3	IL-10	IL-13	TARC	TNF- *α*
−	NM	0.02	0.03	0.05	0.016	0.656	0.152
+	CT	1	1	1	1	1	1
+	CsA	0.202	0.404	0.621	0.386	0.12	0.255
+	RAE (450 mg/kg)	0.462	0.499	0.577	0.284	0.487	0.401
+	RAE (45 mg/kg)	0.62	0.725	0.504	0.763	0.944	0.625
